# Analyzing causal relationships in proteomic profiles using CausalPath

**DOI:** 10.1016/j.xpro.2021.100955

**Published:** 2021-11-23

**Authors:** Augustin Luna, Metin Can Siper, Anil Korkut, Funda Durupinar, Ugur Dogrusoz, Joseph E. Aslan, Chris Sander, Emek Demir, Ozgun Babur

**Affiliations:** 1 Department of Cell Biology, Harvard Medical School, Boston, MA 02115, USA; 2 Department of Data Science, Dana-Farber Cancer Institute, Boston, MA 02215, USA; 3 Broad Institute of Harvard and MIT, Cambridge, MA 02142, USA; 4 Computational Biology Program, Oregon Health and Science University, 3181 SW Sam Jackson Park Rd, Portland, OR 97239, USA; 5 Department of Bioinformatics and Computational Biology, The University of Texas MD Anderson Cancer Center, Houston, TX 77030, USA; 6 Computer Science Department, University of Massachusetts Boston, 100 William T. Morrissey Blvd, Boston, MA 02125, USA; 7 Computer Engineering Department, Bilkent University, Ankara 06800, Turkey; 8 Knight Cardiovascular Institute, Oregon Health and Science University, 3181 SW Sam Jackson Park Rd, Portland, OR 97239, USA; 9 Department of Molecular and Medical Genetics, Oregon Health and Science University, 3181 SW Sam Jackson Park Rd, Portland, OR 97239, USA; 10 Pacific Northwest National Laboratories, 902 Battelle Blvd, Richland, WA 99354, USA

**Keywords:** Bioinformatics, Proteomics, Systems biology

## Abstract

CausalPath (causalpath.org) evaluates proteomic measurements against prior knowledge of biological pathways and infers causality between changes in measured features, such as global protein and phospho-protein levels. It uses pathway resources to determine potential causality between observable omic features, which are called prior relations. The subset of the prior relations that are supported by the proteomic profiles are reported and evaluated for statistical significance. The end result is a network model of signaling that explains the patterns observed in the experimental dataset.

For complete details on the use and execution of this protocol, please refer to Babur et al. (2021).

## Highlights

• A free open-source tool for exploration of proteomic data
• Analysis focuses on causal relationships revealed by proteomic changes
• Network visualization and publication-quality figures of CausalPath results

## Graphical abstract

**Figure:**
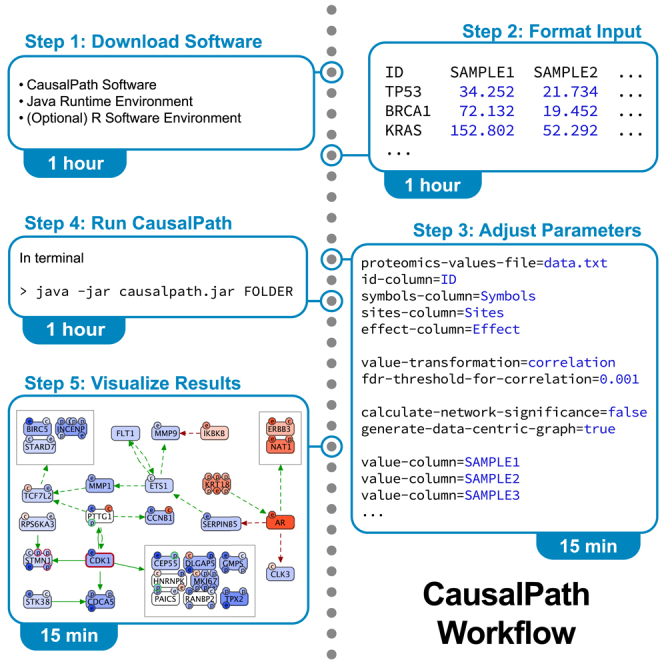

## Before you begin

### Overview

CausalPath maps proteomic and other molecular profiles to pathway information from the Pathway Commons database (Rodchenkov et al., 2020; Babur et al., 2021; Barsi and Szalai, 2021) and other resources, focusing on causality among correlated measurements. This mapping generates a model of molecular signal flow that is consistent with both the profiling data and the published literature. Results are presented as a network model with links to information found in the Pathway Commons database. This analysis mimics aspects of the “manual” exploration of the scientific literature that researchers perform when they review their experimental data. CausalPath allows the systematic exploration of hundreds of thousands of curated mechanisms, which would be infeasible to do manually.

The protocol below describes the specific steps for the CausalPath analysis of a proteomics study that quantified changes in >3000 phosphopeptide levels using mass spectrometry, related to the initialization and progression of platelet GPVI signaling (Babur et al., 2020; Aslan, 2021). We have also used similar protocols for the analysis of high-throughput mass spectrometry and reverse-phase protein array (RPPA) screens from National Cancer Institute's Clinical Proteomic Tumor Analysis Consortium (CPTAC) and The Cancer Genome Atlas (TCGA) programs, respectively (Babur et al., 2021).

**CRITICAL:** A prerequisite to following this protocol is basic knowledge for 1) exploring a computer file system and 2) running commands through a command-line terminal.

### Download and install Java, CausalPath, and ChiBE

**Timing: <1 h**

CausalPath can be either run through its web server (causalpath.org), or locally from its JAR file. While the former option does not require local installation of any special software, the latter option provides more capability and is therefore included in this protocol. A local run of CausalPath requires the Java software environment, which is freely available. CausalPath is available as a Java Archive (.jar) for download on GitHub; this tutorial presents instructions for CausalPath 1.2.0. Additionally, users interested in ChiBE (Chisio BioPAX Editor) for visualization can go to the ChiBE website for software download (Babur et al., 2010, Babur et al., 2014).

1. Download and install the Java 8 installation package from Oracle (oracle.com/java) or OpenJDK (adoptopenjdk.net); installers are provided for Windows, Linux, and MacOS. CausalPath has been tested with both Java 8 and 11 from Oracle or OpenJDK; it has been tested more thoroughly with Oracle Java 8.
2. Download the causalpath.jar file (github.com/PathwayAndDataAnalysis/causalpath/releases); version 1.2.0 is used for this protocol.
3. (Optional) Download and install the ChiBE software (Chisio BioPAX Editor; github.com/PathwayCommons/chibe) for the visualization of results. Supported onLinux and Windows platforms.

***Alternatives:*** Visualization of results can be done using the CausalPath website (causalpath.org), and the Newt tool (http://web.newteditor.org); no download or installation is required in either case.

***Note:*** This protocol provides instructions for visualization with the CausalPath website and Newt.

**CRITICAL:** CausalPath can be considered installed and ready to run once 1) the input files and the casualpath.jar are located on the user's computer as detailed in the section "Run CausalPath" and 2) Java has been installed.

### Download and install the R environment and readxl/stringr packages (to reformat source data if needed)

**Timing: <1 h**

This step installs the R environment and readxl/stringr packages to extract and reformat example data from Excel spreadsheets.

4. R is a free software environment for statistical computing and graphics. It runs on Linux, Windows and MacOS. Download from (r-project.org). The current protocol was tested using R version 3.6.2.
5. Install the “readxl” R package with the command:

install.packages("readxl")

install.packages("stringr")

***Note:*** This step is not necessary if your data is already in the necessary input format for CausalPath.

## Key resources table

**Table:** 

REAGENT or RESOURCE	SOURCE	IDENTIFIER
**Software and algorithms**		

CausalPath	Babur et al., 2021; https://github.com/PathwayAndDataAnalysis/causalpath/releases	1.2.0
ChiBE (Chisio BioPAX Editor)	Babur et al., 2010; https://github.com/PathwayCommons/chibe	2.1
Java	https://adoptopenjdk.net/	11
R	https://cran.r-project.org/	3.6.2
Readxl	https://cran.r-project.org/web/packages/readxl/index.html	1.3.1
Stringr	https://cran.r-project.org/web/packages/stringr/index.html	1.4.0

## Materials and equipment

### Software

CausalPath is a freely available open-source project hosted on GitHub. The software is written in Java. This protocol uses the R (3.6.2) as well as the readxl (1.3.1), and stringr (1.4.0) packages to reformat example data from an Excel spreadsheet into the necessary plain-text, tab-delimited, tabular input format. A list of software used is in the Key Resources Table.

### Data format

The example input data consists of phosphoproteomic abundance measurements collected from a TMT-SPS-MS3 proteomics study that quantified >3000 phosphopeptides related to the initialization and progression of platelet GPVI signaling (Babur et al., 2020).

The required columns of the input file are labeled with the following names: “ID”, “Symbols”, “Sites”, “Effect”, and “Value”. Column names may differ from their default labels; the configuration file (i.e., parameters.txt) specifies the column names. Below is a description of the contents of each column for a given row.

*ID***:** A unique text identifier for each row in the dataset. Ideally, the ID contains the gene symbols and modification sites if applicable. These IDs will be used within the visualization software. Recommended format SYMBOL_SITE (e.g., G6PD_Y401).

**CRITICAL:** IDs should not contain the characters “|” and “@”. Otherwise, it may interfere with ChiBE visualization.

*Symbols:* The HGNC symbol for the related gene. If there is more than one gene symbol related to a given row, then there should be a single space between each symbol.

*Sites:* If the row contains a phosphoprotein measurement, this column should indicate protein sites that are affected. The format for the site is the one letter capital amino acid abbreviation followed by an integer for the location on the UniProt canonical sequence (e.g., Y401, or S439). In the case of multiple sites, the sites should be separated with a pipe character “|” (e.g., S151|T153); termed a site group. If a row is related to more than one gene symbol, then there should exist a site group for each symbol that is separated with a single space. See “Sites” column for MAPK11/MAPK12 or the EHBP1 entries in the Example Input below.

*Effect:* If the row is a phosphoprotein measurement, this column can contain the effect of the related phosphorylation on the protein activity. Use “a” for activating and “i” for inhibiting phosphorylations. If the effect cannot be classified as activating or inhibiting, then it should be left as blank (preferred) or “c” for “complex effect”. If “c” is used then the row will be removed from possible causes, as these “complex effects” cannot be currently evaluated. If this column is blank for a row, then CausalPath will use data compiled from various resources such as PhosphoSitePlus, Signor, and limited, manual in-house curation (Hornbeck et al., 2019; Licata et al., 2020).

*Value (named “SignedP” in protocol example data):* A numeric value for the row. There can be multiple value columns (with distinct column names) depending on how the measurements are presented. A numeric value may encode various types of information depending on the analysis. Values may represent normalized protein reads, control and test columns, a change in expression levels (e.g., fold change), or p-values that are generated by statistical tests. The type of information stored in value columns is specified in the configuration file (i.e., parameters.txt). In this protocol example, we have a single value column, named “SignedP” that stores signed p-values where the sign indicates the change direction and the value is the multiple-testing-adjusted p-value from a moderated t-test; both are provided in the original dataset.

***Alternatives:*** When a “fold change” is used in the value column, the “value-transformation” parameter should be set to “arithmetic-mean”, and the threshold value should be provided using the “threshold-for-data-significance” parameter.

***Alternatives:*** The column names can be different from those specified here; the configuration file (i.e., parameters.txt) is used to specify the column names. Files for this protocol use the column name “SignedP” as an example of “Value”.

**Table:** Example input

ID	Symbols	Sites	Effect	SignedP
G6PD_Y401	G6PD	Y401	A	-0.0012
MAP3K7_S439	MP3K7	S439		-4.6e-5
FRY_S1955	FRY	S1955	I	7.1e-3
MAPK11_T180_Y182_MAPK12_T183_Y185	MAPK11 MAPK12	T180|Y182 T183|Y185	a	0.80
EHBP1_S426_S432_S436	EHBP1	S426|S432|S436		-4.39e-5
…	…	…	…	…

***Note:*** (Babur et al., 2021) describes many additional use cases for CausalPath along with input data files available on Zenodo (zenodo.org/record/4477801).

### Available configuration options and format

A configuration file contains the parameters for a CausalPath analysis. Each parameter in this file should be given in a separate line, in the format “parameter-name = parameter-value”. This protocol will use the following subset of parameters. A complete list of parameters along with additional descriptions is available on the project website (github.com/PathwayAndDataAnalysis/causalpath/blob/master/wiki/InputFormat.md).

**CRITICAL:** The file containing these parameters must have the exact name “parameters.txt”.

• proteomics-values-file: A relative path to the proteomics data file (e.g., “data_causalpath.txt”)
• id-column: Name of the column with IDs (e.g., “ID”)
• symbols-column: Name of the column with gene symbols (e.g., “Symbols”)
• sites-column: Name of the column with sites (e.g., “Sites”)
• effect-column: Name of the column with effects (e.g., “Effect”)
• value-column: Name of the column with values; use only when a single set of values is being considered (e.g., “SignedP”)
• value-transformation: A transformation applied to the values in the value-column. Use of “signed-p-values” denotes p-value for each row has been pre-calculated and that negative p-values indicate a downregulation and vice versa. (e.g., “signed-p-values”)
• threshold-for-data-significance: A threshold value for selecting significant data; format “thr-val data-type” (e.g., “0.1 phosphoprotein”)
• color-saturation-value: In the network visualization, this parameter determines which value maps to the most intense upregulation and downregulation colors. When p-values are used in the analysis, this parameter applies to −log(p-value) (e.g., “10”)
• calculate-network-significance: If value is "true", a p-value for activation and inhibition of proteins in the prior network will be calculated through permutation testing.
• permutations-for-significance: Number of iterations to use in permutation testing. (e.g., “10,000”)
• fdr-threshold-for-network-significance: The false discovery rate for network significance calculations for the downstream activity detection. (e.g., “0.1”)
• use-network-significance-for-causal-reasoning: After calculation of network significances, setting this value to “true” makes CausalPath use the inferred activities in causal reasoning.
• show-all-genes-with-proteomic-data: Whether to include disconnected genes in the results. If the value is “true”, any protein that is represented among causal priors and has significant value change will be represented on the result network, even if they are disconnected.
• gene-activity: Assign a specific activity or inactivity to a gene; format: gene name and one letter code for activity (“a”) or inactivity (“i”). (e.g., “BRAF a”)

### Data reformatting code

The following R code will extract data for use with CausalPath from the Supplementary File 2 from the recent publication on GPVI signaling (Babur et al., 2020). This code should be saved as “phospho_mass_spec_data_extract.R” to be used as part of this protocol.

library(readxl)

library(stringr)

# READ DATA ----

# Supplementary File 2 from

https://www.sciencedirect.com/science/article/pii/S0006497120799349

dat <- read_excel("1-s2.0-S0006497120799349-mmc2.xlsx",

    sheet="Cond #2 TiO2 Brief", skip=11)

# INITIALIZE RESULTS DATA.FRAME ----

reformatted_dat <- data.frame(ID=character(0), Symbols=character(0),

       Sites=character(0), Effect=character(0),

       SignedP=numeric(0), stringsAsFactors=FALSE)

# EXTRACT VALUES ----

pb <- txtProgressBar(min=1, max=nrow(dat), style=3)

for(i in 1:nrow(dat)) {

 setTxtProgressBar(pb, i)

 gene_symbol <- dat$`UniProt Gene Name`[i]

 # Format as 1-letter amino acid abbreviation and site number (e.g., Y7)

 position <- dat$`Site List`[i]

 positions <- str_split(position, "; ")[[1]]

 sites <- paste(positions, collapse="|")

 sites_id <- paste(positions, collapse="_")

 id <- paste0(gene_symbol, "_", sites_id)

 t1 <- data.frame(ID=id, Symbols=gene_symbol,

     Sites=sites, Effect="",

     SignedP=sign(dat$logFC[i])∗dat$FDR[i],

     stringsAsFactors=FALSE)

 reformatted_dat <- rbind(reformatted_dat, t1)

}

# The original and reformatted data should have the same row count

stopifnot(nrow(dat) == nrow(reformatted_dat))

# SAVE RESULTS ----

write.table(reformatted_dat, "data_causalpath.txt", sep="\t",

   row.names=FALSE, quote=FALSE)

## Step-by-step method details

### Format data

**Timing: 1 h**

The code below will run quickly (< 1 min). Modification of the R code for particular user datasets is likely to be more time-consuming.

1. The code can be run in R with the command:

source("phospho_mass_spec_data_extract.R")

**CRITICAL:** The purpose of this step is to produce input files in the format as described in the “Data Format” section. Users are free to choose alternative methods for conducting this step; see “Alternatives.” This step is not necessary if your source data is already formatted for CausalPath.

***Alternatives:*** The data reformatting step can be done in many different ways. This protocol uses the R environment as an example. Users are encouraged to format source data in the CausalPath input format in the manner they are most comfortable whether it be through the use of any programming language or an alternative file editing method they prefer.

**CRITICAL:** The R code and input Excel spreadsheet must be in the same directory.

**CRITICAL:** Reformatted data will be produced in the file “data_causalpath.txt”. Modifications to the code should result in a data file with a data format as described above.

***Alternatives:*** The provided R code converts one of the sheets (named “Cond #2 TiO2 Brief") in the original xlsx file. An alternative is to include multiple sheets in the same conversion for more complete analysis.

### Adjust parameters

**Timing: <15 min**

Make modifications to the “parameters.txt” as necessary

2. Modify, add, or remove parameters in the “parameters.txt” file while keeping the format of each line as “parameter-name = parameter-value”

proteomics-values-file = data_causalpath.txt

id-column = ID

symbols-column = Symbols

sites-column = Sites

effect-column = Effect

value-transformation = signed-p-values

value-column = SignedP

threshold-for-data-significance = 0.1 phosphoprotein

color-saturation-value = 10

calculate-network-significance = true

permutations-for-significance = 10000

fdr-threshold-for-network-significance = 0.1

use-network-significance-for-causal-reasoning = true

show-all-genes-with-proteomic-data = false

gene-activity = GP6 a

***Note:*** Users reproducing this protocol do not need to make modifications to the parameters.txt above.

***Note:*** Pre-formatted data and configuration files, as well as result files, are at this link (zenodo.org/record/5311589).

### Run CausalPath

**Timing: 1 h**

Run the CausalPath algorithm

3. Ensure that the "causalpath.jar", "data_causalpath.txt", and "parameters.txt" files are organized in the directory structure as shown below.

***Note:*** For a single analysis, both the input files data file (e.g., “data_causalpath.txt”) and configuration file (i.e., “parameters.txt”) can be put in a directory by themselves, as shown above. For multiple analyses of the same data, the data can be placed in the parent directory and reused for the different analyses.

***Note:*** Results will be produced in the same directory as the “parameters.txt” file.

4. Run the CausalPath analysis from a command line terminal
  java -jar causalpath.jar gpvi_mass_spec
  ***Note:*** Results will be produced in the same directory as the input files. The directory structure of a successful CausalPath run will appear as follows:
  Description of all possible output files is available on the project site (github.com/PathwayAndDataAnalysis/causalpath/blob/master/wiki/OutputFiles.md). The principal files are as follows:
  a. causative.sif: A Simple Interaction Format (SIF) network representation for identified causal relations is visualized. Used by Newt and ChiBE visualization.
  b. causative.format: This file complements causative.sif and provides styling (e.g., color information) for the SIF. Used by Newt and ChiBE visualization.
  c. causative.json: Result network in JSON format, used by the webserver for visualization.
  d. results.txt: Details of each inference made by the algorithm. This file is useful when further computation is planned on the CausalPath results.

### Visualize phosphoproteomic network using causalpath.org

**Timing: <15 min**

CausalPath results can be visualized and explored using the project website (causalpath.org).

**Figure 1:**
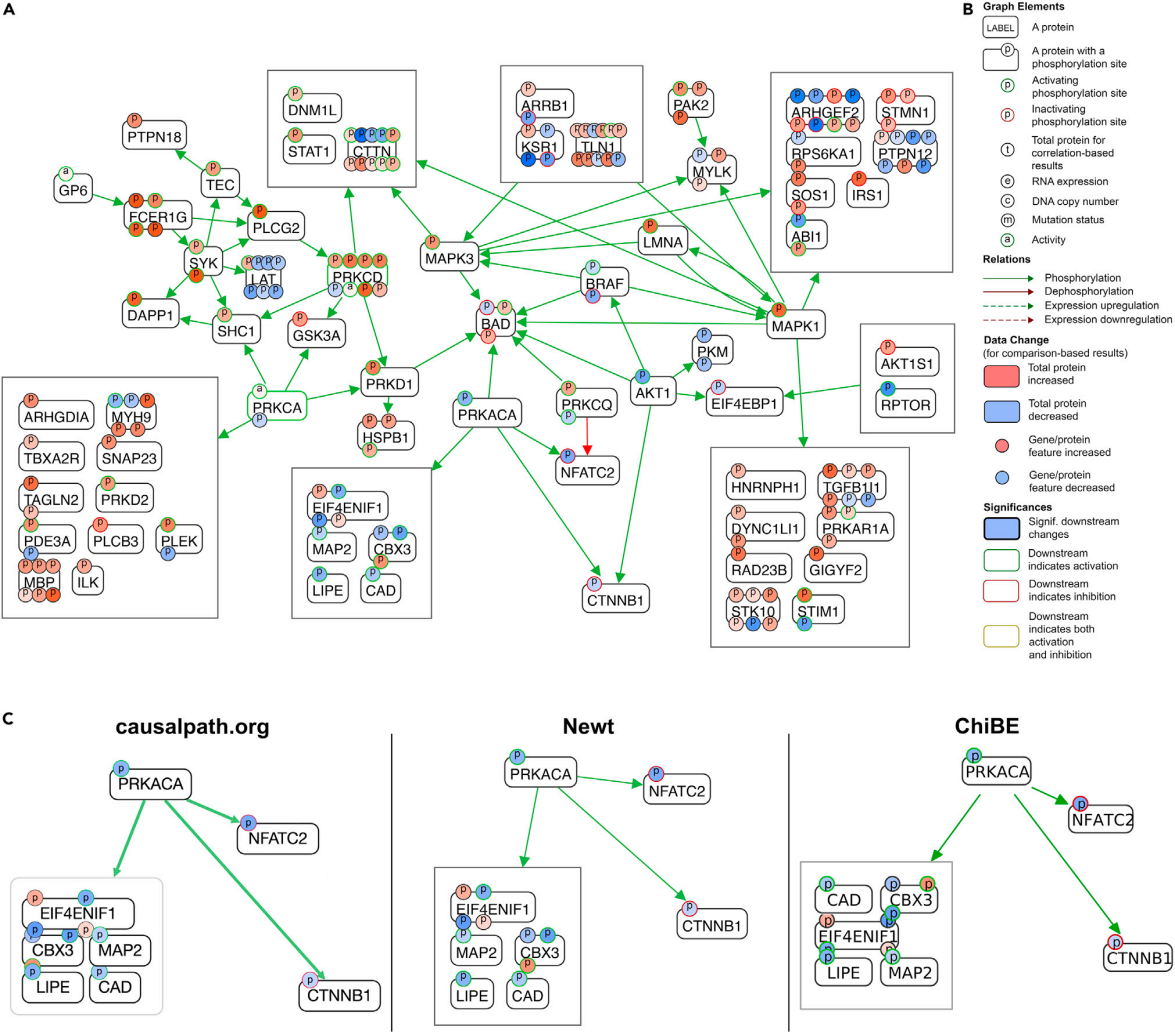
CausalPath output (A) Resulting causal network. (B) Legend for graph notation for causal explanations; see (Babur et al., 2021) for a detailed explanation. (C) Render comparison of CausalPath output using alternative tools.

5. On causalpath.org, click “Visualize results from previous analysis”
6. Select the folder containing the result files (see the “Run CausalPath” section for details) or a parent folder that contains multiple result folders. Figure 1 visualizes the output of CausalPath.

**CRITICAL:** A folder must be selected and not individual files

7. Accept the browser request to upload files if prompted
8. Once the upload completes and the visualization page loads, the next step is to double-click the folder name from the left-hand sidebar

***Note:*** Users can click the “Show legend” button for a guide to the visualization.

***Note:*** Users can drag nodes to reposition them and click on phosphorylation sites to learn their positions.

***Optional:*** The complexity of large networks is managed (i.e., simplified) through the “Topology grouping” feature. “Topology grouping” groups nodes that have the same graph topology. This feature can be turned off by clicking the “Topology grouping” checkbox.

### (Alternative) visualize results using the Newt pathway editor

**Timing: <15 min**

CausalPath results can alternatively be visualized and explored using the Newt Pathway Viewer & Editor (web.newteditor.org) (Balci et al., 2020).

9. Click File → Import → SIF then select the “causative.sif” to import the network

10. Click File → Import → SIF Style the select the “causative.format” to import the network colorization
  ***Optional:*** “Topology grouping” can be turned on to simplify large networks. This is done by clicking the “Map” tab on the right-hand sidebar then clicking the “Enable Topology Grouping of SIF Nodes” checkbox.
  ***Alternatives:*** Users can also use ChiBE as a visualization tool and instructions are provided on the ChiBE project page (github.com/PathwayCommons/chibe).
  ***Note:*** The text here summarized the three options for visualization of results; Figure 1C shows a render comparison between the tools. The comments below may guide user decisions in selecting the best tool for their needs.
  a. The CausalPath site
    i. Provides a legend to help explore the networks
    ii. Does not provide an image export feature; take a screenshot if needed
    iii. Visualization is done as a client-side browser operation that does not require sending files to the server
  b. Newt/ChiBE
    i. Exports high-resolution vector graphics useful for publication-quality images
    ii. Only requires the upload of resulting networks.
  c. ChiBE
    i. Can be run locally
    ii. Runs on Linux and Windows.

## Expected outcomes

The set of 1) resulting output files and 2) the network graphs both as images and interactive graphs (via the visualization tools) are outputs of the protocol.

## Limitations

Currently, CausalPath cannot analyze phosphorylation sites that have complex effects. This can occur in situations where the current understanding in the literature points to both an activating and inhibiting function for a particular site.

Separately, for this protocol example, we have provided R code to extract and reformat input data into the CausalPath format. Often data suitable for CausalPath is found in *ad hoc* formats in spreadsheets, and within a single protocol, it is not possible to account for all possible variations in which this data may be found.

CausalPath uses several information resources to help infer the resulting network and results are limited to the contents of these resources (Babur et al., 2021). Two specific limitations that result from the pathway databases we utilize include: 1) that this project is solely to examine datasets from human cells and 2) there are no interactions for protein isoforms in these resources, currently.

With version 1.2.0 presented in this protocol, users can only analyze phosphorylation post-translational modifications (PTMs).

## Troubleshooting

### Problem 1

The following error during Run CausalPath can be caused by a data formatting issue.

Exception in thread "main" java.lang.ArrayIndexOutOfBoundsException: Index

4 out of bounds for length 3

  at

org.panda.causalpath.resource.ProteomicsFileReader.readVals(ProteomicsFile

Reader.java:136)

  …

### Potential solution

Ensure that your input data format matches the description in this protocol. In addition, ensure that the column names used in “parameters.txt” match those in the input data file. Missing data can be represented as “NaN” entries.

### Problem 2

The following error during Run CausalPath can be caused by a misspelling in the “parameters.txt”:

Exception in thread "main" java.lang.IllegalArgumentException: No enum

constant org.panda.causalpath.data.DataType.PHOSPHOPRTEIN

  at java.base/java.lang.Enum.valueOf(Enum.java:240)

  at org.panda.causalpath.data.DataType.valueOf(DataType.java:8)

  …

### Potential solution

Ensure that the assigned values in “parameters.txt” entries match the available options that are described at the wiki page (github.com/PathwayAndDataAnalysis/causalpath/blob/master/wiki/InputFormat.md) and spelled correctly.

### Other problems

We have thoroughly tested the CausalPath software, but other problems may occur.

### Potential solution

For other technical problems or identified bugs, users can post their questions on the GitHub repository (github.com/PathwayAndDataAnalysis/causalpath/issues/new).

## Resource availability

### Lead contact

Further information and requests for resources should be directed to and will be fulfilled by the lead contact, Özgun Babur (ozgun.babur@umb.edu).

### Materials availability

This study did not generate new unique reagents.

## Data Availability

All necessary data and source code for this protocol is freely and publicly available on GitHub. The CausalPath Java Archive (.jar) can be downloaded from: (github.com/PathwayAndDataAnalysis/causalpath/releases). Pre-formatted data, configuration, and R code files are at the following link (zenodo.org/record/5311589)
